# Does cleaning of venipuncture site with alcohol affect blood ethanol concentration?

**DOI:** 10.1016/j.heliyon.2024.e31517

**Published:** 2024-05-17

**Authors:** Deniz Kılıç, Adeviyye Aksoy, Ramazan Sivil, Taylan Kılıç, Ali Avcı, Mustafa Keşaplı, Güzin Aykal

**Affiliations:** a Department of Emergency Medicine, University of Heath Sciences, Antalya Training and Research Hospital, Antalya, Turkey; b Department of Emergency Medicine, Antalya City Hospital, Antalya, Turkey; c Department of Medical Biochemistry, University of Heath Sciences, Antalya Training and Research Hospital, Antalya, Turkey

**Keywords:** Laboratory testing, Diagnostics, Alcohol, Errors, Pre-analytical phase

## Abstract

**Background:**

It is important to accurately determine the blood ethanol concentration (BEC) to ensure appropriate diagnosis and treatment of patients in the emergency department (ED) and protect their legal rights. This study aimed to determine whether sterilization of venipuncture site with ethanol, which is frequently used in practice in the ED would affect BEC.

**Methods:**

Venous blood samples were collected by two consecutive techniques from 94 individuals who were admitted to the ED, had an indication for BEC measurement, and volunteered to participate in the study. The reference technique involved applying 3 cc of 10 % povidone-iodine solution to a gauze pad, cleaning the right arm antecubital region, and performing phlebotomy. The index technique used 3 cc of alcohol-based antiseptic (89 % ethanol) on another gauze for cleaning the left arm antecubital region. Both techniques allowed the antiseptic to air-dry for 30 s before phlebotomy. Two blood sample tubes per patient were sent to the laboratory, and BEC were measured using the alcohol dehydrogenase enzymatic method.

**Results:**

94 patients were included in the study. The mean age was 37.8 years (±15.7), with 77 % (n = 72) of them were male. The median BEC levels measured by both the reference and index techniques were 2 mg/dL (IQR: 0.97–16.25) and 2 mg/dL (IQR: 0.90–15.22), respectively, with no significant statistical difference (p = 0.536). 72 (77 %) of the patients had a BEC level below the legal driving limit of 20 mg/dL. Bland-Altman analysis, performed on these patients, revealed a small negative bias, −0.116 mg/dL with a standard deviation of 1.13 mg/dL. The upper and lower limit of the agreement was 2.092 and −2.323 respectively.

**Conclusion:**

In patients with a BEC level of less than 20 mg/dL, using ethanol-containing antiseptics before blood sampling does not lead to erroneously elevated BEC levels.

## 1 Introduction

Alcohol abuse and alcoholism is a major health problem in current societies. Blood ethanol concentration (BEC) estimation may be necessary in the emergency department (ED) if there is suspicion of concomitant alcohol intake in medical conditions such as stupor, coma, trauma, and polypharmacy, as well as in forensic cases [1].

Guidelines by international organizations, particularly World Health Organization (WHO), suggest that errors occur mostly in the preanalytical phase, and they provide the measures to avoid these errors accordingly [2,3]. A national guideline on venous blood collection came out in 2015 by Turkish Biochemical Society Preanalytical Phase Working Group so as to prevent erroneous blood collection and ensure accurate test results. These guidelines are aimed at ensuring that blood tests are conducted correctly, eliminating preventable errors that occur during blood collection, and benefiting staff who serve as phlebotomists by relying on evidence-based sources [4,5].

Alcohol-impregnated swabs are usually used to clean the skin before phlebotomy [2]. Although this procedure has been proven to have no value in the cleansing of the venipuncture site and needle penetrations of the skin without skin preparation has not shown to cause any local or systemic infection, most guidelines continue to recommend the use of alcohol swabs [3,4].

As stated in both international and Turkish guidelines, for general purposes, the venipuncture site should be cleansed with 70 % ethanol and allowed to dry before phlebotomy [2]. If a blood culture is to be taken, after wiping with 70 % ethanol, it should be further cleansed using an iodine solution applied in a circular motion, allowing the iodine compound to dry [2,3]. However, if BEC measurement is needed, it is recommended to wipe the skin with 1/100 diluted benzalkonium chloride or povidone-iodine solution [2,3,5]. Also, contamination of blood with povidone-iodine can cause a false increase in potassium, phosphorus, and uric acid values. In the presence of such a concern, cleaning with alcohol may take precedence [[5], [6], [7]].

Alcohol can be absorbed through the intact skin of adults and may slightly affect BEC by values between 0.04 and 0.18 mg/dL, but this increase is not physiologically relevant [8]. When the phlebotomist fails to allow the alcohol to dry for at least 30 s before venipuncture, there is an increased risk of the needle tip becoming contaminated with liquid alcohol from the skin [9]. Some measurable analytes in the blood may have considerable forensic implications, such as the average BEC that limits driving across Europe when over 50 mg/dL. Of note, the BEC driving limit for candidate drivers is capped at 0 mg/dL in most European countries and 80 mg/dL in the United Kingdom. In Turkey, the BEC limit is established at 20 mg/dL for candidate drivers, motorcyclists, and commercial vehicle operators, while for personal car drivers, it is set at 50 mg/dL [10].

Many factors may affect the estimation of BEC of an individual. Phlebotomists worldwide commonly believe that using an alcohol-based swab to cleanse the venipuncture site before blood sampling can affect BEC. However, there is no definite evidence supporting this theory. Besides, in Turkey, Courts of Law are compelled to consider this information, and in many cases, the defense attempts to exploit it for profit or to sow seeds of doubt. Despite certain local forensic regulations prohibiting the use of alcohol-based cleaning methods for the venipuncture site before blood sampling, this may be overlooked or deemed impractical in busy ED.” [11]. Studies report that the use of alcohol-based antiseptics when drawing blood is common [7,9,12].

Accurate measurement of BEC is of great importance in both vital and forensic matters, thereby arising the question whether BEC obtained by using an alcoholic antiseptic causes falsely high values.

In this study, we aimed to investigate whether the use of an ethanol and ethanol-free antiseptic before venous blood collection in a busy and non-ideal environment such as the ED affects the measured BEC.

## 2 Methods

### 2.1 Study design and setting

The current study was conducted in the ED of a tertiary care hospital by prospectively recruiting the patients requiring BEC measurement for any reason. All subjects signed a written consent to participate in the study, which was conducted in accordance with the Declaration of Helsinki, under the provisions of relevant local legislation.). If a patient was suspected of intoxication, researchers contacted them 24 h later to verify their informed consent. Patients who did not recall providing consent were excluded from the study.

The present study was approved by the local Ethics Committee (University of Health Sciences, Antalya Training and Research Hospital Clinical Practice Ethics Committee, approval no: 15/23 date: October 01, 2020).

### 2.2 Study patients

#### 2.2.1 Inclusion criteria

- Patients requiring BEC measurement for any reason.
- Being over 18 years of age

#### 2.2.2 Exclusion criteria

- Patients denied giving inform consent
- Patients less than 18 years of age

### 2.3 Blood sample collection

Initially, the antecubital region of the right arm was sterilized using an ethanol-free antiseptic containing a 10 % povidone-iodine solution, followed by the collection of a venous blood sample. Subsequently, the left antecubital region was cleansed using an antiseptic with 89 % ethanol, and another venous blood sample was obtained using a tourniquet. In both procedures, the antiseptic applied before venipuncture was allowed to air-dry for 30 s. The blood specimens obtained were dispensed into labeled blood tubes, indicating the corresponding arm for each patient. Given the potential legal ramifications for the research participants, the results yielded from the samples provided from the arm disinfected with povidone iodine (ethanol-free solution) were meticulously documented in official records to uphold and safeguard the participants' rights. The identical protocol was adhered to for the collection of all blood samples.

Batiqon® (Batiqon 100 mL (mL), Alfa Temizlik Medikal, Istanbul, Turkey) containing 10 % povidone-iodine solution was used as the alcohol-free antiseptic. This solution was maintained in a plastic container provided by the hospital, which dispensed a volume of 3 cubic centimeter (cc) with each manual pump. 3 cc povidone-iodine solution was applied to a 4 cm^2^ area on the sterile gauze swab. Following the cleansing of the right antecubital region, the antiseptic was permitted to air-dry before obtaining a blood sample. This approach was designated as the reference technique.

A solution containing 89 % ethanol (Cas No: 64-17-5) and 11 % excipients (Skinman Soft Protect; Ecolab Europe GmbH Richtistr, Wallisellen, Switzerland) prepared for routine blood collection at the hospital was used as an ethanol antiseptic. One pump (3 cc) of ethanol was applied to a 4 cm^2^ area on the sterile gauze to sterilize the left antecubital area. Following the cleansing of the left antecubital region, the antiseptic was permitted to air-dry before obtaining a blood sample. This approach was designated as the index technique.

For each venipuncture, a 19 Gauge straight needle and a 10 mL syringe (Setecoject 10 mL Luer, disposable syringe, Set Medikal San. Ve Tic. A.Ş., İstanbul, Turkey) were used. For each patient, 2 blood samples, one for the reference technique and one for the index technique, were delivered to the laboratory via gel vacuum tubes (5 mL, Vacusera, Izmir, Turkey).

Each venipuncture application was standardized according to national guidelines [2]. When blood sampling was required, the procedure was performed by any nurse on duty in the ED at the time. In all cases, the blood tube was filled to the level marked by the manufacturer. Contact between the needle and ethanol cotton wool swab was also carefully avoided throughout the blood sampling process. The blood collection process and waiting times were inspected by an independent observer, thus ensuring standardization. Immediately after sampling, the blood sample tubes were gently inverted and mixed 4 times. The phlebotomists performing the sampling procedure were blinded to the aim of the study. But they could potentially discriminate between the solutions because of the color difference of the solutions. The individuals performing the BEC measurements were also blinded to the method of venipuncture site cleaning.

No complications, such as bleeding, subcutaneous ecchymosis, restricted joint mobility, arthritis, arterial damage, venous thrombosis, thrombophlebitis, cellulitis, or sepsis, were observed in any participant.

### 2.4 Methods of BEC measurement

Venous blood samples collected in 5 mL gel vacuum tubes were centrifuged at 4000 rpm (1968×*g*) for 10 min at 6 °C. The serum ethanol levels were then analyzed using the alcohol dehydrogenase (ADH) enzymatic method with the Improgen kit (Improgen Diagnostic, Istanbul, Turkey) on the Beckman Coulter AU680 analyzer (Beckman Coulter, Indianapolis, USA).

### 2.5 Data analysis

Study data were analyzed with Statistical Package for the Social Sciences (SPSS) version 26 (SPSS Inc., Chicago, IL, USA) and The Jamovi Project (2023) Version 2 [Computer Software, www.jamovi.org]. While the numerical data were presented as mean ± standard deviation for demographic displays and Bland-Altman analysis, it was presented as median (IQR: Interquartile Range) in two independent group comparisons since not distributing normally. Mann-Whitney *U* test was performed for the comparison of two independent groups. Bland-Altman analysis was used to determine the limits of agreement and acceptability between the two measurements. The Shapiro-Wilk test was employed for the normality analysis.

A sample size of at least 64 participants per group was estimated ahead of the study, considering a medium effect size (d: 0.5) with a 5 % type-1 error and 80 % power in both directions.

All the hypothesis was constructed as two tailed and an alpha critical value of 0.05 was accepted as significant.

### 2.6 Variability in study data

**Fig. 1:**
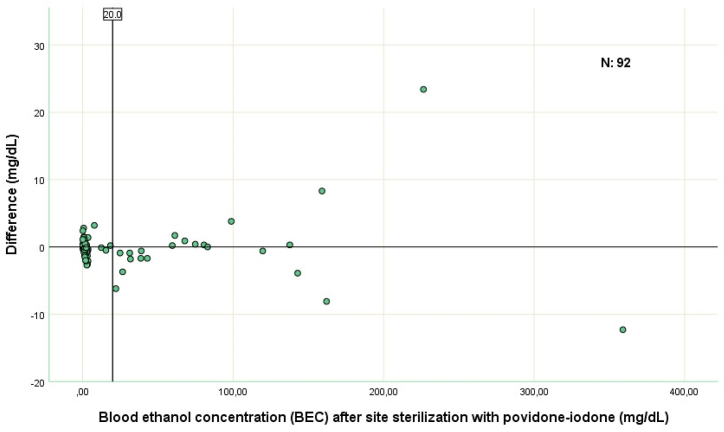
The differences between the results of both measurement methods can be seen. When calculating the difference between the two methods, the BEC measured by the index technique was subtracted from the BEC measured by the reference technique (povidone-iodine). mg: milligram, dL: deciliter.

As the BEC soared, variability of the study data augmented which was likely secondary to the method used to measure the BEC levels (Fig. 1). This variability also led to a flaw in normality of the study data. Bland-Altman analysis is crucial in establishing the agreement level of two procedures and more robust when used with the data being normally distributed. But the data of 94 patients recruited initially did not normally distribute. However, the data of the patients with a BEC level of <20 mg/dL distributed normally, which is also the legal limit for all drivers except personal car drivers in Turkey. So, Bland-Altman analysis was performed by the patients with a BEC level of less than 20 mg/dL.

## 3 Results

Ninety-four patients were recruited into study with a mean age was 37.8 years (±15.7) and %77 (n = 72) of them were male. The median BEC levels of the study patients measured by both the reference and index techniques were 2 mg/dL (IQR: 0.97–16.25) and 2 mg/dL (IQR: 0.90–15.22), respectively, with no statistical difference (p = 0.536, Mann-Whitney *U* test) (Table 2).

**Table 1:** Socio-demographic characteristics and outcomes of study patients.

Variables	n = 94
**Age (year)** Mean ± SD **Gender, n (%)** Male Female **Ethanol values (mg/dL), mean** ± **SD, min-max** Reference technique- povidone-iodine solution Index technique- Ethanol solution Difference between measurements	37.8 ± 15.7 72 (77) 22 (23) 23.9 ± 55.1 (0.1–359) 23.9 ± 55.3 (0–347) 0.12 ± 3.31 (−12.3 - 23.4)

Abbreviations: SD: standard deviation, min-max: minimum-maximum.

**Table 2:** Demographic features of study patients and patients with a BEC of less than 20 mg/dL.

Variables (n)	All Patients (n = 94)	Patients with a BEC of less than 20 mg/dL (n = 72)
Mean Age (SD)	37.8 (15.7)	37,4 (16,2)
Gender, n (%) Male Female	72 (77) 22 (23)	52 (72) 20 (28)

**Mean/Median BEC**	**Mean (SD)**	**Median (IQR)**	**Mean (SD)**	**Median (IQR)**
**Sterilized with povidone-iodine**	**23.94 (55.1)**	**2 (0.97–16.25)**	**2.23 (3.07)**	**1.60 (0.8–2.47)**
**Sterilized with ethanol**	**23.85 (55.3)**	**2 (0.90–15.22)**	**2.11 (3.21)**	**1.35 (0.6–2.1)**
**P Value**		**0.536**		**0.333**

Abbreviations: BEC: blood ethanol concentration; SD: standard deviation; IQR: interquartile range.

**Fig. 2:**
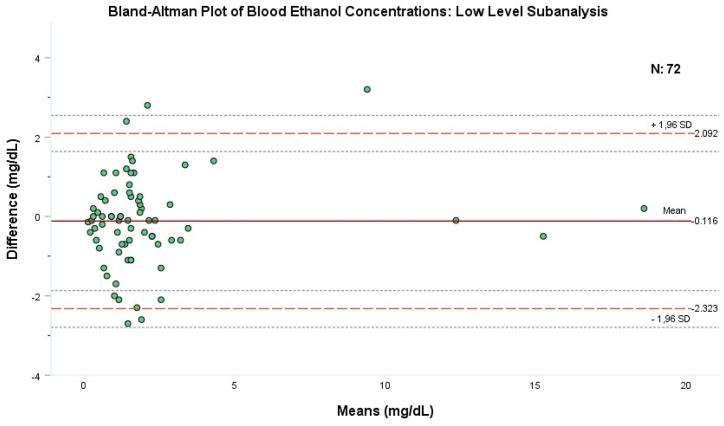
The X-axis shows the mean of two measurements obtained by the study methods, while the Y-axis shows the difference between both measurement methods. The straight line in the middle part of the chart shows the average of measurement differences, the dashed line at the top shows the upper limit of the Acceptable Limit (LOA), and the dashed line at the bottom of the chart shows the lower LOA. The thin dots above and below each baseline provide the 95 % Confidence Interval for each LOA.

Seventy-two (77 %) of the patients had an ethanol level below the legal driving limit of 20 mg/dL (Table 1). Bland-Altman analysis was performed by those 72 patients whereby 52 (72.2 %) of them were male with a mean age of 37.4 (±16.2) years (Table 2). The mean and median BEC levels provided through the reference technique was 2.23 mg/dL± 3.07 (%95CI: 1.51–2.95) and 1.6 mg/dL (IQR 0.8–2.47), respectively, while it was 2.11 mg/dL ± -3.21 (%95CI: 1.38–2.87) and 1.35 mg/dL (IQR 0.6–2.1), respectively, for the index technique (Table 2), with no statistical difference (p = 0.536, Mann-Whitney *U* test). Bland-Altman analysis, in those 72 patients with BEC level of less than 20 mg/dL, showed a small negative bias with a mean difference of −0.116 mg/dL and a standard deviation of 1.13 mg/dL between the two methods. The upper limit of the agreement was 2.092 and the lower limit of the agreement was −2.323. As shown in Fig. 2, most data points cluster around zero difference and fall within the limits of agreement.

## 4 Discussion

The ED is a complex, chaotic atmosphere and limited research has been conducted globally in the ED to determine how venipuncture site sterilization techniques are linked to the BECs. So, it is essential to conduct a robust study for determining the association of cleaning technique of venipuncture site and the BEC. The sample-collecting environment of the present study represents the entire population and typical conditions of an ED which has not been studied before.

The present study showed no physiologically significant BEC difference between alcohol containing and alcohol-free antiseptic solutions for sterilization of venipuncture sites of the ED patients requiring BEC measurement for any reason and had BEC levels of <20 mg/dL. Bland-Altman analysis also showed a slight difference between two methods with clinically acceptable agreement limits.

Research inquiring into the use of ethanol as a sterilization solution on the venipuncture site for the measurement of the BEC is limited and insufficient. Yet, when taking a glance at the studies up to now, false positive higher BEC levels are commonly related to not allowing the venipuncture site to dry, contact of the blood sampling needle with the alcohol-soaked swab and removement of the needle out of the skin while the blood sample aspiration is in progress [9,[13], [14], [15]]. In our study, we let the sterilization solutions dry for 30 s, thereby hampering the procedure related flaws.

We observed that as the BEC levels increased (especially above 80 mg/dL), the variations between the results of the BEC increased significantly (Fig. 1). Since the blood sampling techniques were standardized, it was thought that the high variations seen at high BEC's could be related to the laboratory measurement method of the blood samples. The laboratory kit used in the present study works on the ADH enzymatic method. This was also compatible with the manual of the laboratory kit, as stated in the manual, there can be a 1 mg/dL and 1.6 mg/dL deviations in a result of 40.4 mg/dL and 151.1 mg/dL of the BEC levels, respectively (Appendix 1). On the contrary, this variation does not exist in patients with BEC levels of <20 mg/dL. Although it may be considered normal to see high deviations in high BEC levels, we were unable to explain the variations in the BEC levels between techniques, exactly. It is unclear whether the difference is due to ethanol containing sterilization solutions, error in the laboratory kit or enzymatic method used for BEC measurement, or unknown factors that we have not yet considered. The laboratory kit we used in the study operates on the ADH enzymatic method to measure BEC. It is controversial that when the ADH method is used for the measurement of the BEC, elevated LDH (Lactate Dehydrogenase) or blood lactate levels can result in false positive higher BEC levels [16,17]. Since we did not routinely assess LDH and blood lactate levels in study patients, we were unaware of whether we had encountered such situations.

### 4.1 Limitations

There are some limitations to this study. Firstly, the study data was not distributed normally which, in turn, limited the statistical analysis to patients with a BEC level of less than <20 mg/dL. Although there were 72 patients with BEC level of less than 20 mg/dL which exceeds the estimated sample size, extrapolation of outcomes to patients with a BEC levels of over 20 mg/dL is debatable.

Secondly, the method of participant selection might introduce selection bias, as only those seeking medical attention in the ED were included, potentially excluding individuals who might have different characteristics or alcohol consumption patterns.

Another limitation to the present study is the usage of a laboratory kit utilizing ADH enzymatic method for the measurement of BEC. This may interact with LDH or lactate, thereby causing false positive elevations of BEC results. Although enzymatic measurement and gas chromatography (GC) were reliable methods for measuring BEC values, we were unable to utilize GC method [18]. So, we were unable to explain the BEC variations between two techniques.

Iodine and alcohol containing antiseptic solutions have different colors, which can prevent phlebotomists from visually distinguishing between them. The study's methodology would have been more robust if the solutions had been the same color.

Sample size needed for the present study was established as per the comparison of two independent groups rather than the Bland-Altman analysis. Hence, sample size of the study data (n = 72) used for Bland-Altman analysis is underpowered.

## 5 Conclusion

Using ethanol containing antiseptics before blood sampling is not related to erroneous elevations in BEC levels in patients with a BEC of less than 20 mg/dL. Interactions between the ethanol containing antiseptics and BEC levels in patients with higher BECs (>20 mg/dL) require further research.

## Sources of support

None.

## Funding

This research did not receive any specific grant from funding agencies in the public, commercial, or not-for-profit sectors.

## Data availability statement

For the readers who asking for further data available please directly contact corresponding author via e-mail (ade.aksoy@gmail.com).

## CRediT authorship contribution statement

**Deniz Kılıç:** Writing – review & editing, Supervision, Investigation, Data curation, Conceptualization. **Adeviyye Aksoy:** Writing – review & editing, Writing – original draft, Validation, Supervision, Methodology, Investigation, Data curation, Conceptualization. **Ramazan Sivil:** Validation, Software, Methodology, Formal analysis, Data curation. **Taylan Kılıç:** Formal analysis, Data curation, Conceptualization. **Ali Avcı:** Formal analysis, Data curation, Conceptualization. **Mustafa Keşaplı:** Writing – review & editing, Writing – original draft, Supervision. **Güzin Aykal:** Investigation, Conceptualization.

## Declaration of competing interest

The authors declare that they have no known competing financial interests or personal relationships that could have appeared to influence the work reported in this paper.
