# Supplementary material for: Does cleaning of venipuncture site with alcohol affect blood ethanol concentration?
Source: Heliyon. 2024 May 17;10(10):e31517. doi: 10.1016/j.heliyon.2024.e31517 (PMC11133929; doi:10.1016/j.heliyon.2024.e31517)
Supplement: Multimedia component 1 [file mmc1.pdf]

iMPROGEN Diagnostic Solutions

ETHANOL

Reagent for in vitro quantitative determination of ETHANOL in human serum or plasma

IN VITRO DIAGNOSTIC USE

CE IVD

support@improgen.com

INTENDED USE

Reagent for in vitro quantitative determination of ETHANOL in human serum or plasma on spectrophotometers or Clinical Biochemistry analyzer listed below.

SUMMARY AND BACKGROUND OF THE CLINICAL UTILITY [1-2-3-4-5]

Ethanol (ethyl alcohol) is a widely used and often abused chemical substance. The main pharmacological action of ethanol is the depression of CNS. When consumed with other CNS depressant drugs, ethanol has a potentiating or synergistic depressant effect. The mechanisms of depressant action of ethanol are complex and not fully understood, but likely deal with enhancement of main inhibitory neurons and impairment of excitatory neurons.

PRINCIPLE OF THE METHOD

Ethanol is oxidized by enzyme Alcohol Dehydrogenase (ADH) in the presence of NAD. Absorbance increase per unit time, due to formation of NADH, is proportional to the concentration of sample ethanol and can be monitored at 340 nm.

REAGENT COMPOSITION

R1:

- Buffer (pH: 9,0) 300 mmol/l

R2:

- Buffer (pH: 6,6) 40mmol/l
- NAD ≥10 mmol/l
- ADH ≥200 kU/L

SAFETY PRECAUTIONS

For professional in vitro diagnostic use only.
Do no pipette by mouth. Do not ingest. Harmful if swallowed.
Use protection. (Glasses, gloves, gown etc.)
It is recommended that all specimens or reagents of biological origin should be considered potentially infectious. Practice the normal precautions required for handling all laboratory materials in accordance with OSHA's Bloodborne Pathogens Standards.
Causes serious eye irritation. If in eyes rinse cautiously with water for several minutes and get medical advice.
Causes skin irritation. If on skin wash with plenty of water.
Material safety data sheet available for professional users on request.
Dispose of contents/container according to national/international regulations.

REAGENT HANDLING

Follow the expiration date. Do not use reagent after the expiration date.
Do not run assay by using reagents with different lot number.
Do not compound reagent with any kit and do not add any materials inside the container.
If any air bubbles present in the reagent remove before use.
Discard the reagent if it has developed gross turbidity, change in color, precipitate or other evidence of microbial growth or contamination.

REAGENT PREPARATION, STABILITY AND STORAGE

Ready for use.
Store refrigerated at 2-8 °C.
Shelf life unopened at 2-8 °C until the expiration date printed on the label.
Components are stable once opened until the expiration date marked in the label if they are stored well closed and care is taken to prevent contamination during their use.
Once opened and onboard refrigerated 21 days.

SPECIMEN COLLECTION AND PREPARATION

Heparinized or EDTA- plasma, and serum ine specimen are recommended.
Collect serum using standard sampling tubes.
Ethanol is stable in serum or heparinized plasma;

For 1 days at 15-25 °C
For 3 days at 2-8 °C.

Ethanol is volatile! Immediately after use, close carefully the standard dropper.
Protect from light and avoid repeated freeze.
Centrifuge samples containing precipitate before performing the assay

INTERFERENCE

In serum, no interference was observed by the presence of:

Hemoglobin	≤ 500 mg/dl
Bilirubin	≤ 33mg/dl
Lipids	≤ 1200 mg/dl
Ascorbic acid	≤ 50mg/dl

In urine, no interference was observed by the presence of:

Hemoglobin	≤ 500 mg/dl
Glucose	≤ 1000 mg/dl
Urea	≤ 4600 mg/dl
Creatinine	≤ 630 mg/dl

ASSAY PROCESS

Materials provided: Ethanol Reagent
Materials required but not provided: General laboratory equipment
Procal Ethanol Calibrator, Procon Ethanol. Spectrophotometer with thermostatic cuvette holder or biochemistry autoanalyzer in the list.
The frequency of routine calibration is 7 days.
If there is a change in reagent lot number or major maintenance performed on the analyzer or if control results fall outside acceptable range recalibration is required.
A level of an appropriate quality control should be run every 24 hours to verify the accuracy of the measurement procedure.

© 2018 ImproGen Diagnostic Ltd. All rights reserved

ETHANOL

Reagent for in vitro quantitative determination of ETHANOL
in human serum or plasma

IN VITRO DIAGNOSTIC USE

CE IVD support@improgen.com

MANUEL PROCEDURE

Assay type	: Endpoint
Wavelength	:340 nm
Optical light path	: 1 cm
Temperature	: 37 °C
Direction	: Increase
Zero adjustment	: Reagent blank
Sample/Calibrator	:50 µl
R1	:1000 µl
R2	:250 µl

Mix R1 and Sample and incubate 5 min at +37°C. Then add R2. Mix, after 90 seconds read the absorbance against distilled water by incubating 37°C. Perform other two readings at 60 seconds intervals.

REFERENCE RANGE

1. 30 - 120 mg/dl euphoria, diminution of attention and control
2. 120 - 250 mg/dl excitement, reduced perception, increased reaction times
3. 250 - 400 mg/dl confusion, disturbed vision, muscular incoordination
4. > 400 mg/dl unconsciousness, possible death

This range is given for orientation only; each laboratory should establish its own reference range

MEASURING RANGE

Linearity: The method is linear up to 600 mg/dl.
At Higher concentrations, dilute the sample 1+1 with %0.9 NaCl and multiply the result by factor 2.

The limit of detection: 3,0 mg/dl.

Precision:

Within run

Sample	Mean (mg/dl)	SD	% CV
Sample 1	40,4	0,5	1,24
Sample 2	151,1	0,84	0,56

Between days

Sample	Mean (mg/dl)	SD	% CV
Sample 1	40,8	0,79	≤8,1
Sample 2	151,5	2,32	≤5,8

QUALITY CONTROLS

The control intervals and limits must be adapted to the individual laboratory and country-specific requirements. Values obtained should fall within established limits. Each laboratory should establish corrective measures to be taken if values fall outside the limits.
A level of an appropriate quality control should be run every 24 hours to verify the accuracy of the measurement procedure
It is recommended to use IMPROGEN ETHANOL Kit with controls below

ProConN Ethanol ETH0011 1x5 ml
ProConN Ethanol ETH0021 1x1 ml

CALIBRATION:

The frequency of routine calibration is 7 days.
If there is a change in reagent lot number or major maintenance performed on the analyzer or if control results fall outside acceptable range recalibration is required.

It is recommended to use IMPROGEN ETHANOL Kit the calibrator below

ProCal Ethanol 1 mL – ETH0511
ProCal Ethanol 1 mL – ETH0521

BIBLIOGRAPHY

1. F. Ishizawa, K. Miyata, S. Misawa, A handy and simple apparatus for the determination of ethanol in blood, Natl. Res. Inst. Police Sci. 38 (1985) 25–28.
2.A.W. Jones, Measurement ethanol in salive with QED: enzymatic test device: comparison of results with bloodand breath-alcohol concentrations, J. Anal. Toxicol. 19 (1995) 169–174
3. F. Moriya, Y. Hashimoto, Application of the TriageTM panel for drugs of abuse to forensic blood samples, Jpn. J. Legal Med. 50 (1996) 50–56.
4. A.H. Wu, S.S. Wong, K.G. Johnson, J. Callies, D.X. Shu, We Donn, et al., Evalution of triage system for emergency drugs-of abuse testing in urine, J. Anal. Toxicol. 17 (1993) 241–245.
5. M. Saitou, M. Nakayama, Rapid and simple determination of alcohol in blood using detector tube, Natl. Res. Inst. Police Sci. 36 (1983) 44–47.

COMPATIBLE ANALYZERS LIST (REF)

ImproGen can provide dedicated diagnostic reagents for the Beckman Coulter™ (AU and DxC Series), Abbott™(Architect, Aeroset Series), Siemens™(Advia and Dimension Series), Mindray™ BS Serisi (BS2000, BS2000M, BS800, BS400, BS380), Snibe™(Biossays Series), Tokyo Boeki™ Biolise Series, Erba™ (XL Series), Dirui™ (CS Series), Randox RX series, Hitachi™, and Konelab™ All Series range of chemistry analysers providing you with freedom of choice from an independent manufacturer

SYMBOL KEYS

IVD In Vitro Diagnostic Use Only	R1 Reagent 1	Biological Hazard	Keep Away From Direct Sunlight
LOT Batch Number	R2 Reagent 2	Manufacturer	CONTENTS Contents
REF Order Number	R3 Reagent 3	EC REP Authorized Representative	Temperature Limit
SN Serial Number	Caution! Refer enclosed document	Prescription Use Only	CE Mark
Consult Instruction For Use	Σ Total	Expiration Date	Liquid Format

MANUFACTURER

Improgen Diagnostik Kimya San. Tic. Ltd. Şti | İkitelli O.S.B İsdök San. Sit. 2 Blok No:1 Başakşehir / ISTANBUL
T: +90(212) 809 00 09 F: +90 (212) 809 00 01
www.improgen.com info@improgen.com support@improgen.com

© 2018 ImproGen Diagnostic Ltd. All rights reserved
